# Sri Lanka Pilot Study to Examine Respiratory Health Effects and Personal PM_2.5_ Exposures from Cooking Indoors

**DOI:** 10.3390/ijerph13080791

**Published:** 2016-08-05

**Authors:** Michael J. Phillips, Emily A. Smith, Paul L. Mosquin, Ryan Chartier, Sumal Nandasena, Katherine Bronstein, Myles F. Elledge, Vanessa Thornburg, Jonathan Thornburg, Linda M. Brown

**Affiliations:** 1 RTI International, 3040 Cornwallis Road, Research Triangle Park, NC 27709, USA; mosquin@rti.org (P.L.M.); rchartier@rti.org (R.C.); kbronstein@rti.org (K.B.); melledge@rti.org (M.F.E.); thornburg@rti.org (V.T.); jwt@rti.org (J.T.); 2 RTI International, 701 13th St NW, Suite 750, Washington, DC 20005, USA; emilysmith@rti.org; 3 National Institute of Health Sciences, Ministry of Health, Kalutara 12000, Sri Lanka; sumalnandasena@gmail.com; 4 RTI International, 6110 Executive Boulevard, Suite 902, Rockville, MD 20852, USA; lindabrown@rti.org

**Keywords:** asthma, biomass, cookstove, indoor air pollution, respiratory, Sri Lanka

## Abstract

A pilot study of indoor air pollution produced by biomass cookstoves was conducted in 53 homes in Sri Lanka to assess respiratory conditions associated with stove type (“Anagi” or “Traditional”), kitchen characteristics (e.g., presence of a chimney in the home, indoor cooking area), and concentrations of personal and indoor particulate matter less than 2.5 micrometers in diameter (PM_2.5_). Each primary cook reported respiratory conditions for herself (cough, phlegm, wheeze, or asthma) and for children (wheeze or asthma) living in her household. For cooks, the presence of at least one respiratory condition was significantly associated with 48-h log-transformed mean personal PM_2.5_ concentration (PR = 1.35; *p <* 0.001). The prevalence ratio (PR) was significantly elevated for cooks with one or more respiratory conditions if they cooked without a chimney (PR = 1.51, *p* = 0.025) and non-significantly elevated if they cooked in a separate but poorly ventilated building (PR = 1.51, *p* = 0.093). The PRs were significantly elevated for children with wheeze or asthma if a traditional stove was used (PR = 2.08, *p* = 0.014) or if the cooking area was not partitioned from the rest of the home (PR = 2.46, *p* = 0.012). For the 13 children for whom the cooking area was not partitioned from the rest of the home, having a respiratory condition was significantly associated with log-transformed indoor PM_2.5_ concentration (PR = 1.51; *p* = 0.014).

## 1. Introduction

Burning biomass in traditional, inefficient cookstoves with poor ventilation results in elevated levels of household air pollution (HAP) and leads to an estimated four million premature deaths per year [1]. It also can lead to lung cancer, acute lower respiratory infections, chronic obstructive pulmonary disease, cataracts, tuberculosis, cardiovascular conditions, adverse perinatal health outcomes, and acute health problems [2,3,4,5,6]. As the primary cook, women may spend more than four hours per day near the cookstove [7]. As a result, they are at much greater risk of developing these potentially life threatening disorders than most other adults in the household. However, children, the elderly, and other adults who spend time near the stove are also vulnerable to the high concentrations of particulate matter (PM) produced by the stoves [8].

Sri Lanka is a small Asian country of approximately 21 million people. Approximately 80% of the population or 17 million people, rely on biomass fuels for meal preparation in their homes [9]. The two most commonly used cookstoves in Sri Lanka are the “Traditional” stove, similar to the three-stone fire but often surrounded by mud or clay, and an improved single-piece clay stove with a more enclosed design called the “Anagi” [10]. Anagi stoves are thought to improve the combustion efficiency of the biomass fuels compared to the Traditional stove, thereby significantly reducing PM exposure from HAP and improving the health of the cook and others in the home [11]. To date, only one peer-reviewed Sri Lankan study has included measurements of PM produced by the two cookstove types, and to our knowledge no studies have measured personal PM HAP exposures directly [12].

From July to September of 2012, a pilot study was conducted in a rural Sri Lankan community that measured personal and indoor (fixed location near the cookstove) concentrations of PM_2.5_ in 53 households. Details about the study design and the results of exposure monitoring are presented in a previous publication [13]. That paper reported that indoor PM_2.5_ concentrations were reduced by 65% when a chimney was present (*p <* 0.001) and by 25% (*p* = 0.054) when an Anagi stove (versus a Traditional stove) was used. Furthermore, personal PM_2.5_ concentrations were slightly higher than indoor PM_2.5_ concentrations at low indoor concentration levels but much lower at high indoor concentration levels, presumably because the cook moved away from the stove when the smoke was intolerable [13]. The current manuscript describes the observed association between self-reported respiratory health conditions of the primary cook, and children residing in her household, and personal and indoor PM_2.5_ concentrations influenced by stove and kitchen area characteristics.

## 2. Materials and Methods

### 2.1. Study Design

This cross-sectional study was conducted in the rural village of Kopiwatta, near Kandy, in central Sri Lanka in 2012. RTI International teamed with the Integrated Development Association (IDEA; a non-profit organization that led efforts to build, promote, and disseminate the Anagi stove throughout Sri Lanka) and an investigator from the Sri Lanka Ministry of Health to collect data and conduct the personal and indoor air pollution measurements. A community facilitator from Kopiwatta, employed by IDEA, led the recruitment effort and screened households to identify nonsmoking households that used either the Traditional or Anagi cookstove. The recruitment effort and purposive sampling yielded 53 households with women who cooked and were willing to participate in the exposure study. For a detailed description of the study design and data collection see Chartier et al. [13].

Prior to data collection, RTI staff trained field technicians to administer the questionnaires, to obtain physical measurements of the homes, and to deploy and download data from the exposure monitors. RTI staff oversaw the first few weeks of data collection to ensure the protocol was being followed and that the exposure monitors were working properly. Regular check-ins occurred throughout the study duration.

For each primary cook, personal and indoor PM_2.5_ concentrations were measured concurrently for two consecutive 24 h periods with MicroPEM™ (RTI International, Research Triangle Park, NC, USA) PM exposure monitors. The cook wore the MicroPEM monitor during waking hours. During the same timeframe, a second MicroPEM monitor was placed near the cookstove to measure “indoor” PM_2.5_ concentrations. This indoor measurement was used as a surrogate for the children’s exposures since they did not wear personal monitors. However, the location of the monitor for this purpose was not ideal since children were unlikely to spend most of their day near the stove, particularly if it was separate or partitioned from the rest of the house. During the 48 h monitoring period, the field team deployed the MicroPEM exposure monitors and recorded stove, chimney, and cooking area measurements and features. They also asked the cook questions about the demographic characteristics of her household and cooking practices [13].

#### 2.1.1. Exposure Data

Other cooking-related characteristics were also examined including: presence of a chimney and location of the cooking area (categorized as: “partitioned from the rest of the home,” “not partitioned from the rest of the home,” or “separate building from living space”). Partitioned from the rest of the home indicates that a door existed between the cooking area and the rest of the home and presumably cooking areas that were not partitioned allowed more smoke throughout the home. For cooks only we additionally investigated number of years cooking and percentage of time shared cooking responsibilities.

#### 2.1.2. Respiratory Health Conditions

The Sri Lanka Ministry of Health investigator administered a health questionnaire to the primary cook about her self-reported respiratory symptoms and those of children aged 17 and younger in each household [13]. Respiratory conditions identified for the cooks were cough, phlegm, “neck whistle” (wheeze), and doctor-diagnosed asthma. A new dichotomous variable, “cook had one or more respiratory conditions”, was formed based on these conditions. It was coded “Yes” if the cook had one or more of these conditions and “No” if the cook had none of these conditions. Two cooks responded “No” to having cough, phlegm, or wheeze; however, their responses to doctor-diagnosed asthma were left blank. In order to code their responses into the new dichotomous variable, it was assumed that these two women did not have doctor-diagnosed asthma (only one cook reported having doctor-diagnosed asthma and she also reported having the other three respiratory conditions). One cook was missing information for all four respiratory conditions and was excluded from the respiratory analyses. Two additional cooks were missing information on personal PM_2.5_ concentrations and were excluded from HAP analysis.

For children, respiratory conditions identified were “neck whistle” (wheeze) and doctor-diagnosed asthma. A new dichotomous variable, “child had 1 or more respiratory conditions”, was derived based on these two conditions. It was coded “Yes” if the child had at least one of these conditions or “No” if the child had neither of these conditions.

### 2.2. Statistical Analysis

Since this is a cross-sectional study, the outcome of interest is the prevalence of respiratory disease. The measure of association used in this analysis is the prevalence ratio (PR) comparing subjects with and without respiratory disease. Log-binomial regression analyses were run separately for cooks and children using the SAS (Version 9.4) GENMOD procedure (SAS Institute, Cary, NC, USA) and R, a software for statistical computing and graphics [14]. Because standard calculations of variances based on the log-binomial regression model assume that responses are independent, we used the repeated statement in SAS to account for multiple children per household.

The PR, 95% confidence interval (CI), and *p* value are presented for each household exposure and demographic variable. One-sided analyses were run for exposure variables with a hypothesized unidirectional relationship with respiratory health outcomes (percentage of time shared cooking responsibilities, type of stove, presence of a chimney, PM_2.5_ exposures). Two-sided analyses were conducted to examine the association between respiratory health outcomes and all additional exposure variables. Personal PM_2.5_ exposures were considered the most representative measure of a cook’s typical daily HAP exposure. Additional variables examined among primary cooks included household size, family monthly income, age, highest level of education achieved, number of years cooking, and location of the cooking area. Indoor PM_2.5_ concentrations from the stationary monitors in the kitchen were used as the exposure variable for children. Additional variables examined among children included the mother’s education level, whether the child was 5 years or older (serves as an indicator of time child spent in the home versus in school), and location of the cooking area.

For the primary cooks, the association of log-transformed 48-h mean personal PM_2.5_ exposure with health outcome was tested using a log-binomial regression model. The model included a linear term for age to control for its possible confounding effect. For children, the presence of a respiratory condition was analyzed using a log-binomial model with a categorical independent variable for location of cooking area. In addition, for children living in homes with a cookstove (i.e., not in a separate building), presence of a respiratory condition was modeled as directly dependent on the 48-h log-transformed mean indoor exposure.

### 2.3. Ethics

On 13 June 2012, the Ethics Review Committee of the Faculty of Medicine, University of Kelaniya, Sri Lanka (Reference number P58/05/2012) approved the study and on 20 June 2012, the Institutional Review Board from RTI International (Identification number 13148) approved the study, which was conducted in accordance with the Declaration of Helsinki. At the end of the study, the cooks were invited to an event where a Medical Officer of Health (i.e., Community Physician) described the adverse health effects of cookstove smoke and ways to improve the indoor air quality of their homes. The Medical Officer of Health, Public Health Midwife, and IDEA staff also presented each cook with a new “Anagi” stove and hot water pot as tokens of appreciation for participation in the study.

## 3. Results

### 3.1. Participant Characteristics

A total of 53 households participated in the pilot study and 35 (66%) of the households included children <18 years old. Table 1 illustrates some of the demographic characteristics, including household size, number of children, and family monthly income. As described in Chartier et al., 2016, all of the cooks were female with a mean age of 47 years, had cooked for an average of 31 years, and most (81%) had finished some secondary schooling. In almost half of the households (*n* = 24 or 45%), another person shared the cooking responsibilities. None of the cooks reported smoking in the past. The average household size was 4.6 members, which is generally consistent with the national average. Most of the households (*n* = 25) had monthly incomes between 15,001 and 25,000 Sri Lankan rupees, an amount well above the poverty level of 3489 rupees, but below the median level of 30,400 rupees across Sri Lanka [15].

### 3.2. Cookstove, Fuels, and Kitchen Characteristics

As presented in Table 1, most households (62.3%) had Anagi stoves, and slightly over half (56.6%) had chimneys. Chimneys were present in 21 (39.6%) of the households with Anagi stoves and nine (17.0%) of the households with Traditional stoves. Most of the cooking areas were partitioned from the rest of the house (45.3%), rather than not partitioned (28.3%) or in a separate building (26.4%). Of households with children, most (48.6%) had cooking areas partitioned from the rest of the house. Dry (purchased) wood was the primary fuel type in nearly all the homes (94%) but other readily available and free fuel sources (e.g., biomass residuals, coconut husks, trash) were also used [13].

### 3.3. Personal and Indoor PM_2.5_ Concentrations

The distribution of indoor and personal PM_2.5_ concentrations is shown in Table 1. Indoor PM_2.5_ concentrations in the cooking area were 200 µg/m^3^ or higher in 13 homes (24.5%) and less than 100 µg/m^3^ in 21 homes (39.6%). The majority of cooks (*n* = 31 or 58.5%) experienced personal PM_2.5_ concentrations less than 100 µg/m^3^, but 20 (37.7%) were exposed to concentrations above 100 µg/m^3^, with six of these (11.3%) exposed to concentrations of 200 µg/m^3^ or higher. These concentrations are considerably higher than the WHO 24-h mean air quality guidelines of 25 µg/m^3^ [16]. The 100 µg/m^3^ threshold used in this analysis has been used in other cookstove research [17].

Table 2 presents the median indoor and personal PM_2.5_ concentrations for cookstove and chimney combinations by the location of the cooking area. Cooks who used Traditional stoves without chimneys experienced substantially higher personal PM_2.5_ concentrations (range = 139–215 µg/m^3^) than those who used Traditional stoves with chimneys (64 µg/m^3^) or those who used Anagi stoves with (range = 47–70 µg/m^3^) or without (range = 83–114 µg/m^3^) chimneys. Personal exposures were lowest (47 µg/m^3^) for women who used Anagi stoves with chimneys in a cooking area not partitioned from the rest of the home; whereas, women who used Traditional stoves without chimneys in a separate building experienced the highest PM_2.5_ concentrations (216 µg/m^3^). The indoor PM_2.5_ concentrations were generally higher than the personal concentrations, particularly when the stove was used without a chimney because the smoke could not escape the structure. For example, the indoor PM_2.5_ concentration (222 µg/m^3^) associated with the use of an Anagi stove with no chimney in a separate building from the living space (*n* = 6), was almost twice as high as the personal PM_2.5_ concentration in the same homes (114 µg/m^3^). This was likely due to the cook moving out of the cooking area (with its higher levels of HAP) when she was not cooking [13].

### 3.4. Health Outcomes for Cooks

Of the 52 cooks, 63.5% self-reported respiratory health conditions. No conditions were reported by 19 (36.5%), 14 (41%) reported one condition, 11 (32.4%) reported two conditions, seven (20.6%) reported three conditions, and one (2.9%) reported all four conditions. The PRs for respiratory conditions by demographic and household exposure factors are shown in Table 3. Using a stove without a chimney was significantly associated with respiratory health outcomes (PR = 1.51, 95% CI = 1.07–+∞). In addition, PRs were higher for cooks who used Traditional stoves (PR = 1.18) and those who used a separate building for cooking (PR = 1.51). When the two highest concentration categories were combined, the PR was significantly elevated for cooks with personal PM_2.5_ concentrations of 100 µg/m^3^ or higher (PR = 1.60, 95% CI = 1.13–+∞; *p* = 0.014).

**Figure 1:**
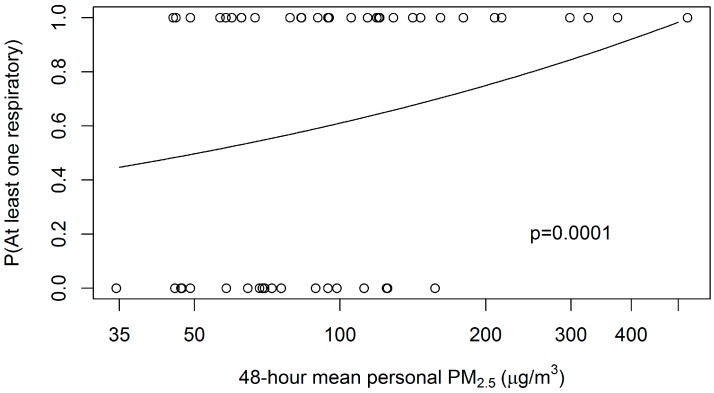
Fitted log-binomial-model probability of any respiratory condition for the primary cook, on 48-h mean personal PM_2.5_ concentration, controlled for age. Plotted points are respiratory condition (0 = none, 1 = any of four).

For the cooks, there was a significant positive association (PR = 1.35; *p* = 0.0001; one-sided) between having at least one reported respiratory health condition and log-transformed mean 48-h personal PM_2.5_ concentration (see Figure 1).

### 3.5. Health Outcomes for Children

Respiratory health information was reported for 63 children (aged <18 years). For two-thirds of the children (*n* = 42), neither respiratory health condition (wheeze (“neck whistle”), or asthma) was reported. Of the 21 children with respiratory health conditions, wheeze was reported for 16 (76.2%) and both wheeze and doctor-diagnosed asthma was reported for five. Of the 35 households in the study with children, respiratory health conditions were reported by both the cook and at least one child in 14 (40%), only by cooks in 11, only by children in four, and by neither cooks nor children in six.

The PRs for respiratory conditions among children by demographic or household exposure variables are shown in Table 4. Overall, there were no associations between having a reported respiratory health condition and demographic variables. However, younger children and children with the least-educated mothers (cooks) were more likely to have a reported respiratory condition.

Among children, presence of a chimney and average indoor PM_2.5_ concentrations were not significantly associated with respiratory health outcomes, but stove type and location of the cooking area were. Children living in households with a Traditional stove were two times more likely (PR = 2.08, 95% CI = 1.21–+∞) to have a respiratory health condition reported compared to those living in a household with an Anagi stove. Children living in a household where the cooking area was not partitioned from the rest of the house were almost two and a half times more likely (PR = 2.46, 95% CI = 1.22–4.95) to have a respiratory health condition reported compared to those where the cooking area was partitioned from the rest of the house.

**Figure 2:**
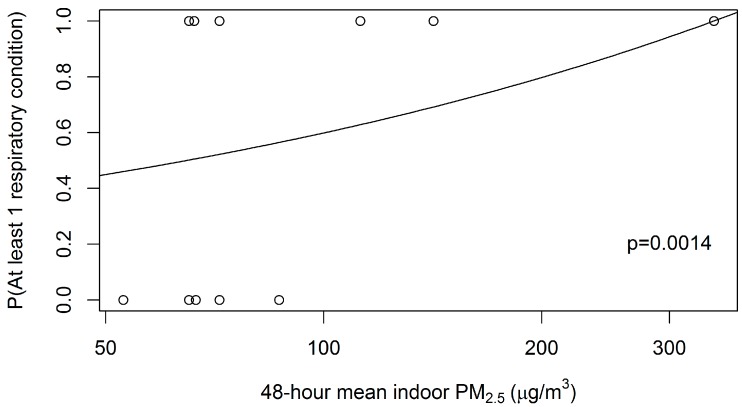
Fitted log-binomial model probability of child having at least one respiratory condition, among 13 children living in homes with kitchens that were not partitioned from the rest of the home. Plotted points are respiratory condition (0 = none, 1 = either of two).

Thirteen children lived in a household where the cooking area was not partitioned from the rest of the house. Of these children, those in homes with Traditional stoves (*n* = 9) were more likely (PR = 1.33; *p* = 0.565) to have a respiratory health condition reported compared to those in households with Anagi stoves (data not shown). Additionally, those living in homes without a chimney were more likely (PR = 1.25; *p* = 0.302) to report respiratory health conditions than those in homes with a chimney (data not shown). For these 13 children, log-binomial modeling of at least one respiratory condition reported dependent on log-transformed indoor PM_2.5_ exposure gave a significant association (PR = 1.51, *p* = 0.014; one-sided). Fitted log-binomial model probabilities are provided in Figure 2.

## 4. Discussion

The results suggest that Sri Lankan cooks and the children living in their homes may experience respiratory health problems due to exposure to high concentrations of PM_2.5_, associated with the physical characteristics of the cooking area and stove. The indoor and personal PM_2.5_ concentrations measured in this study were highest in homes with a Traditional stove and no chimney. The Anagi stove provided a modest reduction in PM_2.5_ concentrations [13]. The 63.5% prevalence estimate of at least one self-reported respiratory condition (cough, phlegm, wheeze, or asthma diagnosis) among the 52 cooks in this study is comparable with other cookstove studies [18]. As expected, cooks with higher personal PM_2.5_ exposures were significantly more likely to report having at least one respiratory health condition. The study findings suggest that cookstove-related characteristics (i.e., location, stove type, and presence of a chimney) have a greater impact on the primary cook’s respiratory health outcomes than household characteristics and the duration of exposure. For example, having one or more respiratory health conditions was not associated with the number of people living in a household or the number of years the woman cooked. However, the PR was significantly elevated if the stove did not have a chimney and non-significantly elevated if the cook used a Traditional stove. It was also non-significantly elevated for cooks who used a stove that was located in a separate building which was typically small and poorly ventilated.

The strongest predictors of reported respiratory health conditions among children were also related to cookstove characteristics. Children in homes with Traditional stoves were significantly more likely to report respiratory problems than those with Anagi stoves; however, the location of the cooking area was particularly important. Children living in a household where the cooking area was not partitioned from the rest of the house were significantly more likely to have reported respiratory health conditions and to have these ailments associated with higher indoor PM_2.5_ concentrations, presumably because the PM was able to reach other rooms in the home where the children spent time. A preferred approach to our use of indoor measurements from a stationary monitor in the cooking area as a proxy for a child’s PM_2.5_ exposure would have been to have each child wear a personal monitor concurrently with the cook and measure all exposures including those outside the cooking area where the child likely spent more time.

Nevertheless, this study was unique because personal exposure measurements of PM_2.5_ were collected for the cooks in addition to indoor measurements; few cookstove research studies measure personal PM concentrations directly [19,20]. Personal PM_2.5_ concentrations were generally lower than indoor PM_2.5_ concentrations, presumably because the cooks move in and out of the cooking area throughout the day. The highest personal PM_2.5_ concentrations were measured in cooking areas located in a separate building from the home, while the highest indoor PM_2.5_ concentrations were measured in a cooking area that was partitioned from the rest of the home.

Although the results of this pilot study are compelling, the sample size was small and the number of homes in each strata (stove type, presence of chimney, and location of stove) was limited (see Table 2), which restricted what could be examined in the analysis. A larger study that includes personal exposure monitoring of the children and/or PM_2.5_ concentration measurements in the bedrooms and other living areas is needed to obtain more definitive results, in order to better understand the impact of cookstove improvements on HAP and the respiratory health of Sri Lankan cooks, children, and other household members. Also due to the purposive sample from a single rural village, the results should not be extrapolated to the general Sri Lankan population, particularly the urban areas where a larger proportion of households use improved cookstoves and clean fuels (e.g., liquid propane gas).

## 5. Conclusions

Our pilot study found that rural Sri Lankan households that used a Traditional stove without a chimney had the highest PM_2.5_ concentrations. In addition, cooks and children from these households were more likely to report respiratory health symptoms. We also found that variables associated with cookstove characteristics (e.g., stove type, presence of chimney, location of cooking area) were stronger indicators of respiratory health outcomes, rather than variables associated with household characteristics and prolonged exposures associated with cooking.

Our results suggest that having a chimney may lower PM concentrations and reduce respiratory health conditions in Sri Lankan cooks. Our findings also suggest that use of an Anagi stove and a partitioned cooking area could reduce the prevalence of respiratory problems in children from HAP. Future studies should include a larger sample size and direct exposure monitoring of the children in the household.

## Figures and Tables

**Table 1:** Distribution of demographic and household exposure indicators among Sri Lankan households.

	All Households		Households with Children	
**Variable**	***N***	**(%)**	***N***	**(%)**
Total	53	(100.0)	35	(100.0)
Household Size				
2*–*3 people	13	(24.5)	3	(8.6)
4*–*5 people	27	(50.9)	23	(65.7)
6*–*7 people	13	(24.5)	9	(25.7)
Number of children (<18 years old) in Household				
0	18	(34.0)	*–*	*–*
1	14	(26.4)	14	(35.9)
2	14	(26.4)	14	(35.9)
3	7	(13.2)	7	(17.9)
Family Monthly Income in Sri Lankan Rupees				
15,000 or less	17	(32.1)	11	(31.4)
15,001–25,000	25	(47.2)	15	(42.9)
25,001 or more	11	(20.8)	9	(25.7)
Type of Stove				
Anagi	33	(62.3)	18	(51.4)
Traditional	20	(37.7)	17	(48.6)
Stove with Chimney				
Yes	30	(56.6)	18	(51.4)
No	23	(43.4)	17	(48.6)
Stove/Chimney Setup				
Traditional stove, no chimney	11	(20.8)	9	(25.7)
Traditional stove with chimney	9	(17.0)	8	(22.9)
Anagi stove, no chimney	12	(22.6)	8	(22.9)
Anagi stove with chimney	21	(39.6)	10	(28.5)
Location of Cooking Area				
Partitioned from rest of home	24	(45.3)	17	(48.6)
Not partitioned from rest of home	15	(28.3)	9	(25.7)
Separate building from living space	14	(26.4)	9	(25.7)
48-Hour Mean Indoor PM_2.5_ Concentrations, µg/m^3^				
Less than 100	21	(39.6)	13	(37.1)
100 to <200	19	(35.9)	12	(34.3)
200 or higher	13	(24.5)	10	(28.6)
48-Hour Mean Personal (cook) PM_2.5_ Concentrations, µg/m^3^				
Less than 100	31	(58.5)	20	(57.1)
100 to <200	14	(26.4)	9	(25.7)
200 or higher	6	(11.3)	5	(14.3)
*missing*	2	(3.8)	1	(2.9)

**Table 2:** Median * PM_2.5_ concentrations (µg/m^3^) for indoor and personal exposures for all households by kitchen location and stove/chimney setup.

	Location of Cooking Area								
	Not Partitioned from the Rest of the Home			Partitioned from the Rest of the Home/Other Room			Separate Building from Living Space		
Stove/Chimney Setup	*n*	Indoor	Personal	*n*	Indoor	Personal	*n*	Indoor	Personal
Anagi stove with chimney	5	66.3	47.2	13	56.0	70.2	3	66.6	62.5
Anagi stove, no chimney	3	141.8	83.1	3	202.2	128.9	6	222.3	114.0
Traditional stove with chimney	5	71.8	64.5	4	109.5	64.0	0	-	-
Traditional stove, no chimney	2	221.5	139.2	4	430.3	160.4	5	417.7	215.7

* Median values in the table represent the median integrated filter concentrations over the 48 h sampling period for all homes with those characteristics (stove/chimney setup).

**Table 3:** Prevalence ratios for respiratory conditions among cooks by demographic and household exposure indicators.

Study Factors	Had 1 or More Respiratory Conditions		PR ^a^ (95% CI)	*p* Value
	*n/N ^^^*	*n* (%) ^P^		
Household Size				
2*–*3 people ^R^	8/12	66.7	1.00	
4*–*5 people	17/27	63.0	0.94 (0.58*–*1.55)	0.821
6*–*7 people	8/13	61.5	0.92 (0.51*–*1.66)	0.790
Family Monthly Income in Sri Lankan Rupees				
15,000 or less ^R^	14/17	82.4	1.00	
15,001–25,000	12/25	48.0	0.58 (0.37*–*0.93)	0.023
25,001 or more	7/10	70.0	0.85 (0.54*–*1.35)	0.490
Age				
35 years or younger ^R^	7/11	63.6	1.00	
36*–*45 years	9/12	75.0	1.18 (0.68*–*2.05)	0.561
46*–*55 years	8/15	53.3	0.84 (0.44*–*1.61)	0.595
56 years or older	9/14	64.3	1.01 (0.56*–*1.83)	0.973
Highest Level of Education Achieved				
None to grade 9 ^R^	14/20	70.0	1.00	
Grade 10	15/26	57.7	0.82 (0.53*–*1.28)	0.385
Grade 11 or higher	4/6	66.7	0.95 (0.51*–*1.80)	0.880
Number of Years Cooking				
Less than 20 years ^R^	6/10	60.0	1.00	
21*–*30 years	10/14	71.4	1.19 (0.65*–*2.18)	0.572
21*–*40 years	11/18	61.1	1.02 (0.54*–*1.90)	0.954
41 years or more years	6/10	60.0	1.00 (0.50*–*2.05)	1.000
Cooks 100% of the time *				
No ^R^	17/24	70.8	1.00	
Yes	16/28	57.1	0.81 (0.57–+∞)	0.153
Type of Stove *				
Anagi ^R^	19/32	59.4	1.00	
Traditional	14/20	70.0	1.18 (0.84–+∞)	0.213
Stove with Chimney *				
Yes ^R^	15/29	51.7	1.00	
No	18/23	72.3	1.51 (1.07–+∞)	0.025
Location of Cooking Area				
Partitioned from rest of home ^R^	12/23	52.2	1.00	
Not partitioned from rest of home	10/15	66.7	1.28 (0.75*–*2.17)	0.365
Separate building from living space	11/14	78.6	1.51 (0.93*–*2.43)	0.093
Mean 48-h Personal PM_2.5_ Concentrations, µg/m^3 1,^*				
Less than 100 ^R^	15/30	50.0	1.00	
100 to <200	10/14	71.4	1.25 (0.92–+∞)	0.120
200 or higher	6/6	100.0	+∞	*–*

^^^ *n =* No. of cases with 1 or more respiratory conditions (cough, phlegm, wheezing (neck whistle), asthma diagnosis) and *N =* total number of cooks with respiratory information (52); ^P^ percentage of cases with 1 or more respiratory conditions; ^R^ reference category; ^a^ prevalence ratio; * one-sided *p* value and CI. ^1^ Note: Personal PM_2.5_ concentrations are available for 50 of the 52 cooks with respiratory information.

**Table 4:** Prevalence ratios for respiratory conditions among children by demographic and household exposure indicators.

Had 1 or More Respiratory Conditions				
Study Factors	*n/N ^^^*	*n* (%) ^P^	PR ^a^ (95% CI)	*p* Value
Mother’s Education Level				
None to grade 9 ^R^	8/21	38.1	1.00	
Grade 10	10/32	31.3	0.82 (0.38*–*1.75)	0.609
Grade 11 or higher	3/10	30.0	0.79 (0.39*–*1.60)	0.509
School-aged (5 years old or older)				
Yes	13/41	31.7	1.00	
No	8/22	36.4	1.14 (0.52*–*2.50)	0.744
Type of Stove *				
Anagi ^R^	8/34	23.5	1.00	
Traditional	13/29	44.8	2.08 (1.21*–*+∞)	0.014
Stove with Chimney *				
Yes ^R^	9/32	28.1	1.00	
No	12/31	38.7	1.45 (0.80–+∞)	0.153
Location of Cooking Area				
Partitioned from rest of home ^R^	8/32	25.0	1.00	
Not partitioned from rest of home	8/13	61.5	2.46 (1.22*–*4.95)	0.012
Separate building from living space	5/18	27.8	1.11 (0.49*–*2.50)	0.799
Mean 48-h Indoor PM_2.5_ Concentrations, µg/m^3^ *				
Less than 100 ^R^	6/24	25.0	1.00	
100 to <200	5/17	29.4	1.18 (0.49*–*+∞)	0.379
200 or higher	10/22	45.5	1.82 (0.92*–*+∞)	0.077

^ *n =* No. of cases with 1 or more respiratory conditions (wheezing (neck whistle), asthma diagnosis) and *N =* total number of children with respiratory information (63); ^P^ percentage of cases with 1 or more respiratory conditions; ^R^ Reference category; ^a^ prevalence ratio; * one-sided *p* value and CI.

## Abbreviations

The following abbreviations are used in this manuscript:

CI	Confidence interval
CO	Carbon monoxide
COPD	Chronic obstructive pulmonary disease
HAP	Household air pollution
IDEA	Integrated Development Association
PM	Particulate matter
PM_2.5_	Particulate matter less than 2.5 mm in diameter
PR	Prevalence ratio
